# Unequal and Invisible: A Feminist Political Economy Approach to Valuing Women's Care Labor in the COVID-19 Response

**DOI:** 10.3389/fsoc.2020.588279

**Published:** 2020-11-06

**Authors:** Michelle Lokot, Amiya Bhatia

**Affiliations:** London School of Hygiene and Tropical Medicine, University of London, London, United Kingdom

**Keywords:** COVID-19, gender, work, race & class, political economy

## Introduction

There is increasing recognition that COVID-19 has exposed and entrenched racial, gender, and class inequalities that have long been neglected across the globe (Ahmed et al., 2020). The intersecting effects of power hierarchies and identities affect women's paid, unpaid, and underpaid labor—particularly their role in providing care—during COVID-19. This includes women's labor in the home, women's roles in the health and social care sectors, and women's role in informal, precarious work including domestic work.

The evidence of women's care labor is unequivocal: globally, women are responsible for the majority (76.2%) of unpaid care work, spending an average of 201 days on unpaid work during a year, compared to 63 days spent on unpaid work by men (International Labour Organization, 2018a). Worldwide, the International Labour Organization (2020a) reports that of the 136 million workers in the health and social care sectors, 70% are women. Around 80% of the world's domestic workers are women (International Labour Organization, 2018b). Migrant domestic workers in particular face additional challenges due to their often-uncertain legal status and limited labor rights.

Women's labor during COVID-19 occurs in a context where structural discrimination already shapes who lives and dies (Chotiner, 2020): racism, gender inequality and unequal and dangerous work have long been linked to adverse health outcomes (Krieger and Smith, 2004). Women's experiences in the workplace are shaped by intersections between gender, age, race, class and migration status (Sheppard, 2011; International Labour Organization, 2020b). These intersections shape who benefits—or suffers—as a result of economic policies, including austerity measures (Bassel and Emejulu, 2014), especially during COVID-19 (Craddock, 2020). The International Labour Organization (2020c) recommendations on the labor market impacts of COVID-19 highlight the combined effects of job losses in hard-hit sectors, women's overrepresentation in the health sector, and higher demands on care labor at home. More recent policy briefs outline the importance of intersectionality in understanding how pre-existing discrimination based on gender and ethnicity in the workplace are exacerbated during COVID-19, particularly in the health and the informal sectors. However, such data are rarely available in low and middle-income countries (LMICs)—further entrenching the invisibility of women's labor. A blindness toward inequalities perpetuates narratives that COVID-19 is “a great equalizer” (Evelyn, 2020). Similarly, reports of men dying at higher rates often fail to specify “which men?,” nor ask “compared to which women?.” Current analyses are thus limited in their ability to critically reflect on power and inequality—which we hope to address in this opinion piece.

We examine three types of women's labor during COVID-19—their care labor at home, labor in health and social care sector, and labor within informal and precarious domestic work. To date, the academic literature on COVID-19 has not addressed these issues using an intersectional lens, particularly for LMICs. For each type of work, we use a feminist political economy lens to explore how women's labor and care work are included or excluded in COVID-19 responses. Such an approach offers a critical analysis of how gender inequality may be sustained through neoliberal economics (Peterson, 2005), and how gender intersects with other power hierarchies and identities such as race and class (Agenjo-Calderón and Gálvez-Muñoz, 2019). It challenges how the economic system perpetuates intersecting inequalities, including how labor is distributed, how labor is valued, and who benefits from the economy and who does not—emphasizing how economic policies are not gender-neutral (Pearson, 2019). We examine emerging evidence on women's labor, highlighting the limitations of COVID-19 responses that do not consider intersections between race, gender and class. We conclude by highlighting concrete steps to ensure protections for women whose labor—and health—are both produced and affected by intersections of race, gender, and class.

## Women's Care Roles in the Home

Historically, feminists have advocated for women's caring roles to be recognized as “work,” but this struggle continues due to entrenched norms that suggest household chores and caregiving are women's “duty” —instead of a result of patriarchy. Hochschild (2012) articulation of the “second shift” where women complete caregiving and household chores in addition to paid employment, is particularly pertinent during COVID-19. Research documents that during COVID-19, women spend more time on home schooling and childcare than men in the United States, United Kingdom and Germany (Adams-Prassl et al., 2020; Oxfam, 2020) research explores connections between living situation and gender, finding that during COVID-19, 70% of women in urban settlements in Nairobi are spending more time on unpaid work. This research also explores intersections between economic status and gender, finding that 50% of poor and marginalized women in the Philippines report increased care labor at home during the pandemic. Other research also documents women's increased care burden, urging equitable distribution of household tasks between women and men (Power, 2020). Notably, most research on women's care labor in the home during COVID-19 tends not to also analyse how other intersecting issues—specifically race and class—affect care burdens at home.

A feminist political economy approach seeks to rectify inequities in division of labor at the household level and increase the value assigned to unpaid work through equitable policies. For example, countries like Sweden have implemented progressive paternity, maternity and shared parental leave policies that encourage men's role in caregiving (Carlson, 2013). Evidence highlights the importance of making it easier for women to continue employment after having children, including through childcare subsidies, and tax credits (Olivetti and Petrongolo, 2017). A recent review of gender-related social protection measures implemented in multiple countries during COVID-19 found only 16 programmes considered women's childcare responsibilities or provided childcare benefits (Gentilini et al., 2020). Other recommendations specific to COVID-19 include ensuring removing the requirement that people actively seek work in order to access unemployment benefits—a particular challenge for those with caregiving responsibilities (Power, 2020). In LMICs, recommendations during COVID-19 include providing food assistance and medication (Bahn et al., 2020).

## Women's Roles in Health and Social Care Sectors

Although women are over-represented in the health sector, female health workers are often paid less than men; data from 104 countries highlights that the gender pay gap in the health sector is 28%. This research also finds that gender norms affect women's representation across different occupations within the health sector, with men working as doctors, dentists and pharmacists while women tend to work in nursing and midwifery roles (Boniol et al., 2019). In contexts like South Africa, narratives on race and gender collide, affirming Black women's roles as nurses–a legacy of colonialism–resulting in Black women's over-representation in care roles (Kalemba, 2020).

During COVID-19, health and social care workers are often most at risk, continuing to provide care to people suffering from COVID-19. In England, deaths reported up to 20^th^ April 2020 indicated more deaths among female health care and social care workers compared to men (Office for National Statistics, 2020). This data is not also available by ethnicity, although a separate report documents higher deaths among Asian and Black women compared to White British women (Public Health England, 2020). Similar to other reports which fail to analyse data across intersecting issues, this report lacks analysis of COVID-19 deaths by occupation and ethnicity.

In contrast, a feminist political economy approach seeks to uncover contributions made by women in these care sectors, while demanding “essential” and “key” workers are appropriately compensated. A review of gender-related measures during COVID-19 found only two programmes that addressed the needs of healthcare workers including providing childcare vouchers (Gentilini et al., 2020). Ensuring personal protective equipment and conducting gender pay gap analysis within the health and social care sectors may be a useful way of ensuring women (who are disproportionately represented in these sectors) are paid what they are worth, and that gaps in pay—which are linked to gender norms and penalties imposed on women for caregiving—are decreased, especially in LMICs.

## Women's Role in Informal and Precarious Domestic Work

The intersections between gender, ethnicity, and class are perhaps most stark in considering women's role in domestic work, including as migrant workers. The labor of domestic workers is often invisible, reinforcing unequal power dynamics between them and the populations they work for. In Malaysia, for example, Indonesian village girls are often cast as “backwards,” contributing labor at low pay for a middle-class group of different ethnicity (Elias, 2010). In Lebanon, migrant domestic workers from countries like Ethiopia are subject to the highly racialised, state-mandated “kafala” system which can lead to exploitation (Pande, 2013). During the COVID-19 pandemic and the challenging economic crisis in Lebanon, economic insecurity among higher-class Lebanese has led to Ethiopian domestic workers being abandoned at their embassy, and denied pay from employers. In other settings, there is growing evidence of other risks faced by domestic workers during COVID-19. Female domestic workers who continue to clean homes and care for sick employers face higher risk of contracting COVID-19 and have limited access to health services (United Nations Economic Commission for Latin America and the Caribbean, 2020). Domestic workers who have lost their jobs during COVID-19 face economic and housing insecurity, as well as the risk of deportation if they are migrant workers (Aoun, 2020). The ILO reports that low- and middle-income countries will suffer the most from COVID-19 disruptions, as they have higher proportions of informal workers who fall outside the remit of social protection programs (International Labour Organization, 2020a).

In contrast, valuing the economic contributions made by women in these precarious forms of care work means encouraging countries to implement policies protecting the rights of domestic workers, to extend social protection schemes, and to ratify existing Conventions. For example, the 2011 Domestic Workers Convention requires that migrant domestic workers receive written contracts, be paid at least the minimum wage, be housed in decent living conditions and be protected from abuse (International Labour Organization, 2016). Unions and migrant labor rights bodies can advocate for domestic workers and ensure they receive information about their rights (International Labour Organization, 2015).

To date, there has not been a coordinated, global response to COVID-19 for workers in the informal economy and formigrant workers (Liem et al., 2020). Their voices are rarely present in policy research (International Labour Organization, 2020b), creating gaps in understanding what is most needed from the perspective of migrant domestic workers. A recent rapid assessment of global social protection measures implemented during COVID-19 found only 16 programmes that addressed the economic challenges faced by informal workers, including providing wage and utilities subsidies, food vouchers, and cash transfers (Gentilini et al., 2020).

## Discussion

COVID-19 exposes multiple fault lines beneath neoliberal, gendered, and racialised policies. This article draws attention to inequalities evidenced in women's labor at home, in the health and social care sectors, and in informal work, drawing attention to how intersections with race, economic status, and other factors enables a deeper understanding of the scope of women's care labor. As policy makers contend with easing lockdown measures and consider the long-term implications of COVID-19 on work, women's labor should be central to research and policy, particularly drawing on the voices of women themselves. Although efforts have been made to make recommendations about gender-responsive social protection during COVID-19, these efforts do not usually consider intersections with ethnicity, race, class, age, and migrant status.

We conclude with four recommendations to recognize women's care labor and address intersecting gendered, racial and class inequalities. First, data that documents women's care roles, disaggregated by sex, race, migrant status, and economic status is essential in understanding the intersecting dimensions to women's care labor both during and after COVID-19. We suggest that researchers and policy-makers focus on three critical spheres in understanding women's disproportionate burden in care roles: the home, the health and social care sector, and within domestic work. Second, workers in the health sector and the informal sector will continue to need protection, with explicit attention required to inequities in access to personal protective equipment, work-site safety, and health care. Third, investment in policies that value women's care labor is urgently needed, including paid sick leave, parental leave policies to encourage men's role in caregiving, reducing gender pay gaps, and ensuring legal protections for domestic workers. Finally, and importantly, to ensure women's perspectives are addressed, the voices of diverse women should be central to developing short- and long-term responses to COVID-19.

## Author Contributions

ML: conceptualized the article, wrote the first draft, reviewed, and edited the article. AB: conceptualized the article, contributed to writing, reviewed, and edited the article. Both authors contributed to the article and approved the submitted version.

## Conflict of Interest

The authors declare that the research was conducted in the absence of any commercial or financial relationships that could be construed as a potential conflict of interest.
